# Single-shot read-out of a superconducting qubit using a Josephson parametric oscillator

**DOI:** 10.1038/ncomms11417

**Published:** 2016-05-09

**Authors:** Philip Krantz, Andreas Bengtsson, Michaël Simoen, Simon Gustavsson, Vitaly Shumeiko, W. D. Oliver, C. M. Wilson, Per Delsing, Jonas Bylander

**Affiliations:** 1 Microtechnology and Nanoscience, Chalmers University of Technology, Kemivägen 9, SE-41296 Gothenburg, Sweden; 2 Research Laboratory of Electronics, Massachusetts Institute of Technology, Cambridge, Massachusetts 02139, USA; 3 MIT Lincoln Laboratory, 244 Wood Street, Lexington, Massachusetts 02420, USA; 4 Institute of Quantum Computing, University of Waterloo, Waterloo, Ontario, Canada N2L 3G1

## Abstract

We propose and demonstrate a read-out technique for a superconducting qubit by dispersively coupling it with a Josephson parametric oscillator. We employ a tunable quarter wavelength superconducting resonator and modulate its resonant frequency at twice its value with an amplitude surpassing the threshold for parametric instability. We map the qubit states onto two distinct states of classical parametric oscillation: one oscillating state, with 185±15 photons in the resonator, and one with zero oscillation amplitude. This high contrast obviates a following quantum-limited amplifier. We demonstrate proof-of-principle, single-shot read-out performance, and present an error budget indicating that this method can surpass the fidelity threshold required for quantum computing.

## Abstract (web summary)

Efficient qubit readout is essential for quantum information technology, which requires sufficient recognition of signal from noise. Here, Krantz *et al*. propose a simplified technique using a Josephson parametric oscillator, demonstrating single-shot readout performance of a superconducting qubit.

## Graphical abstract

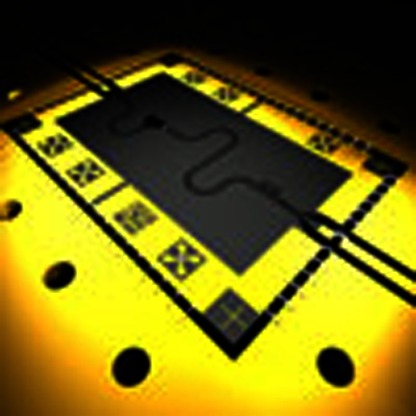

The read-out scheme for quantum bits of information (qubits) constitutes one essential component of a quantum information processor^1^. During the course of a quantum algorithm, qubit-state errors need to be corrected; in many implementations, this is done by quantum error correction, where each operation is based on the outcomes of stabilizer measurements that indicate the qubit errors. The stabilizers must therefore be determined in a single shot—without averaging of the output signals of repeated measurements on identically prepared qubits—with fidelity exceeding approximately 99% (ref. 2).

The commonly used measurement scheme for a superconducting qubit coupled with a linear microwave resonator does not, by itself, offer single-shot measurement performance. The qubit imparts a state-dependent (dispersive) frequency shift on the resonator, which can be determined by applying a probe signal and measuring the reflected or transmitted signal, although only for weak probing, rendering an inadequate signal-to-noise ratio (SNR)^3^,^4^.

Researchers have addressed the problem of insufficient SNR in essentially two ways. One approach is to feed the weak output signal into a following, parametric linear amplifier that adds only the minimum amount of noise allowed by quantum mechanics^5^,^6^,^7^,^8^. Another approach is to insert a nonlinear element into the system and apply a strong drive tone, such that the resonator enters a bistable regime, hence enhancing the detection contrast^9^,^10^,^11^,^12^,^13^.

In this paper, we propose and demonstrate a simplified read-out technique in which a superconducting qubit is directly integrated into a Josephson parametric oscillator (JPO). We map the qubit states onto the ground and excited states of the oscillator, and demonstrate proof-of-concept, single-shot read-out performance (SNR>1). We obtain 81.5% qubit-state discrimination for a read-out time 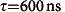; however, from the error analysis, we infer a read-out fidelity of 98.7±1.2%, taking into account known and reparable errors due to qubit initialization and decoherence (17.2±1.2%). A realistically achievable qubit relaxation time, *T*_1_=50 μs, and a Purcell band-pass filter would reduce these errors from 17.2 to <0.5%, as well as shorten the required read-out time to 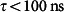. The remaining errors, which are due to the switching events in the oscillator (1.2±0.3%), can be eliminated by improving the data aquisition protocol—see Discussion and Supplementary Note 1. These qubit and detection improvements would bring the read-out fidelity to ≈99.5%.

Our read-out scheme relies on parametric pumping of a frequency-tunable resonator by modulation of its inductance. The pumping amplitude exceeds the threshold for parametric instability, the point above which the resonator oscillates spontaneously, even in the absence of an input probe signal. This instability threshold is controlled by the state of the qubit, whose ground and excited states correspond to the nonoscillating and oscillating states of the resonator, respectively. In our measurement, the oscillating state produces a steady-state resonator field corresponding to 185±15 photons, whose output we can clearly distinguish from the nonoscillating state when followed by a commercial semiconductor amplifier, eliminating the need for a quantum-limited amplifier. Conceptually, this method can yield arbitrarily large contrast due to the parametric instability, and moreover, only requires a pump but no input signal.

This read-out scheme is well aligned with scalable, multi-qubit implementations. Parametric oscillators can be readily frequency-multiplexed^14^ and allow for a simplified experimental set-up (compared with conventional microwave reflectometry) without a separate input port to the resonator or a following parametric amplifier, and consequently, also without additional bulky microwave circulators that would normally route the input and parametric pumping tones. It is also possible to manipulate the qubit via the flux-pumping line only, which further reduces the number of cables and interconnects.

## Results

### The Josephson parametric oscillator

**Figure 1:**
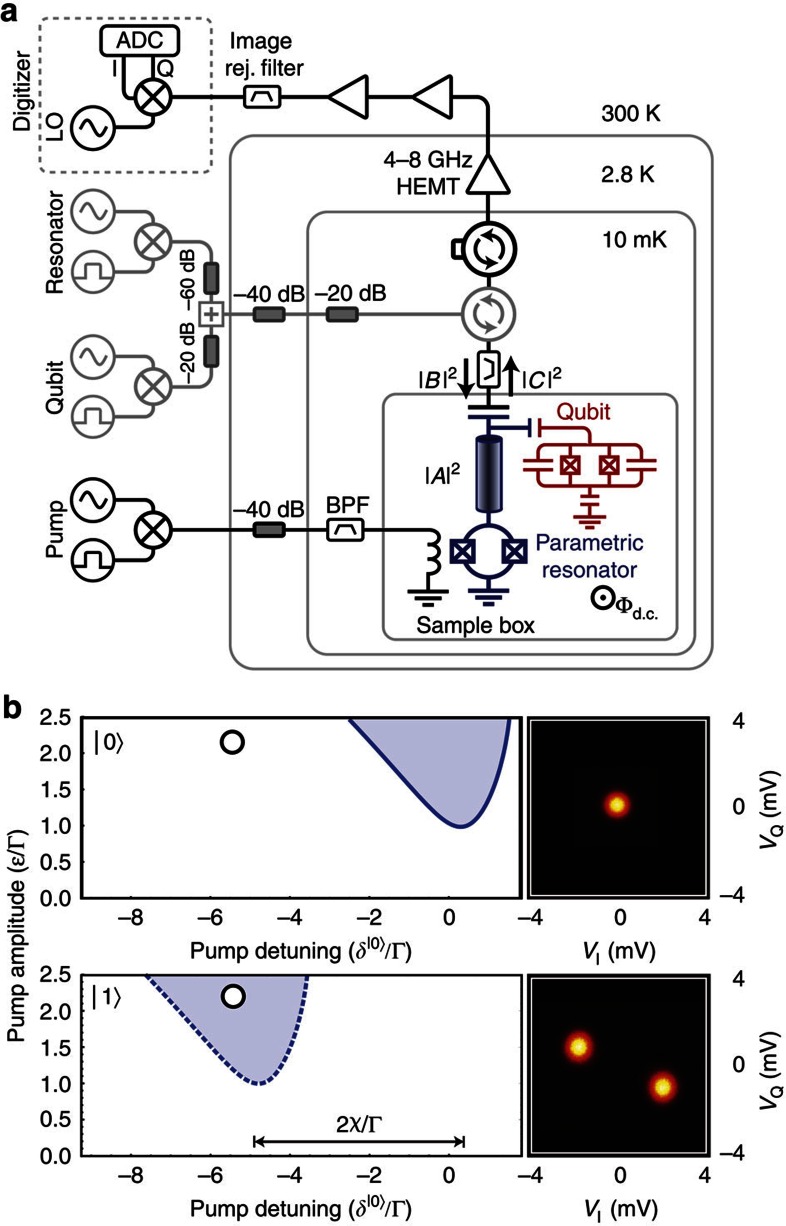
Experimental set-up and read-out mechanism. (**a**) Schematic of the cryogenic microwave reflectometry set-up. The transmon qubit (red) is capacitively coupled with the coplanar waveguide parametric resonator (blue). The input and output flows of photons are denoted |*B*|^2^ and |*C*|^2^, respectively, whereas the number of photons in the resonator is denoted |*A*|^2^. The output signal is acquired using heterodyne detection of the amplified microwave signal. The components drawn in lighter grey are those that are rendered unnecessary by the JPO read-out method, thereby offering a simplified experimental set-up (see text). (**b**) Parametric oscillation regions for the qubit ground state |0〉 (solid blue line) and excited state |1〉 (dashed blue line), respectively. These blue lines represent the instability boundaries, 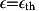, where the number of steady-state solutions to equation (1) changes. The two panels on the right are measured [I,Q]-quadrature voltage histograms of the device output for the pump bias point indicated by the circles, revealing two different oscillator states: outside of the region of parametric oscillations, the resonator is quiet (|*A*|^2^=0). Within the region, the resonator has two oscillating states (|*A*|^2^>0), with a phase difference of *π* radians.

Our device consists of a quarter wavelength (*λ*/4), superconducting coplanar waveguide resonator, shorted to ground in one end via two parallel Josephson tunnel junctions (JJs)—see Fig. 1a. The JJs form a superconducting quantum interference device (SQUID), which acts as a variable Josephson inductance, 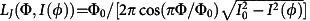, where *I*_0_ is the critical current and Φ_0_ is the flux quantum. This inductance can be controlled by the external magnetic flux through the SQUID loop, Φ(*t*)=Φ_d.c._+Φ_a.c._(*t*), and by the superconducting phase difference across the JJs, *φ*(*t*), via its current—phase relation, *I*(*t*)=*I*_0_ sin*φ*(*t*).

Time-varying modulations of Φ and *φ*—parametric pumping—affect the resonator dynamics, albeit in rather different ways; moreover, the Josephson inductance is indeed both parametric and nonlinear. We explain these differences in the Discussion section below. The resonant frequency of the JPO is parametrically modulated via the magnetic flux, Φ(*t*), which can lead to frequency mixing as well as parametric effects such as noiseless amplification of a signal, frequency conversion and instabilities^6^,^15^,^16^,^17^,^18^,^19^.

The state of the JPO has a rich dependence on several parameters, some of which was studied recently, both theoretically^20^,^21^ and experimentally^7^,^17^,^19^. The equation of motion for the intra-resonator electric field amplitude, *A*, can be written as


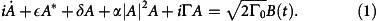


Here 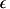 is proportional to the externally applied pump amplitude, Φ_a.c._, which modulates the resonant frequency parametrically at close to twice its value, *ω*_p_≈2*ω*_r_ (degenerate pumping), and *δ*=*ω*_p_/2−*ω*_r_ is the resonator's detuning from half of the pump frequency. The field amplitude, *A*, and its complex conjugate, A*, are slow variables in a frame rotating at *ω*_p_/2, and |*A*|^2^ is the equivalent number of photons in the resonator. The Duffing parameter, *α*, associated with a cubic field nonlinearity, arises from the nonlinear Josephson inductance. The linear damping rate has two components, Γ=Γ_0_+Γ_*R*_, where Γ_0_/2*π*=1.02 MHz is the external damping rate, associated with the photon decay through the coupling capacitor, and Γ_*R*_/2*π*=0.30 MHz is the internal loss rate. The equation's right-hand side represents the input probe signal, such that |*B*(*t*)|^2^ has units of photons per second. The output flow of photons per second, |*C*(*t*)|^2^, is given by 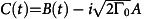.

For low pumping amplitude, below the parametric instability threshold, 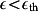, this device works as a phase-sensitive parametric amplifier (JPA) for an input *B*(*t*) at signal frequency *ω*_s_=*ω*_p_/2 (refs 6, 15, 16, 17, 22). Note, however, that we keep *B*(*t*)=0 in the measurements reported here. For a pumping amplitude exceeding the threshold, 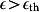, spontaneous parametric oscillations set in—see Fig. 1b and equation (10) in Methods. The resonator field builds up exponentially in time, even in the absence of an input probe signal until it becomes limited by the Duffing and pump-induced nonlinearities and reaches a steady state^17^,^19^.

We connected a transmon qubit capacitively to the resonator^23^—see Fig. 1a. The state of the JPO (oscillating or nonoscillating) can then be controlled by the qubit-state-dependent, dispersive frequency shift, *χ*, which the qubit exerts on the resonator^24^,^25^. When the JPO is being pumped above the threshold for parametric oscillation, with amplitude 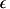 and frequency detuning, *δ*, then a change of qubit state effectively pulls the resonator to a different value of the detuning, outside of the region of parametric oscillations—see Fig. 1b. We denote the qubit-state-dependent detunings by *δ*^|0〉^=*δ*−*χ* and *δ*^|1〉^=*δ*+*χ*. The resulting mapping of the qubit state onto the average number of photons in the resonator provides us with a qubit-state read-out mechanism, which we exploit in this work.

### Characterization of qubit and JPO

The device and cryogenic experimental set-up are depicted in Fig. 1a. The sample is thermally anchored to the mixing chamber of a dilution refrigerator with a base temperature of 10 mK. The parametric *λ*/4 resonator (in blue) is capacitively coupled with the transmission line (*C*_c_=11.9 fF), yielding an external quality factor *Q*_ext_=*ω*_r_/2Γ_0_=2555. A transmon qubit (in red) is also coupled near this end of the resonator.

The resonator output signal is amplified using a 4–8 GHz high-electron-mobility transistor amplifier, with a noise temperature *T*_N_=2.2 K, followed by two room-temperature amplifiers. We detect the outgoing signal using heterodyne mixing. The signal is first downconverted to a frequency (*ω*_RF_−*ω*_LO_)/2*π*=187.5 MHz; then, the [I,Q]-quadrature voltages are sampled at 250 MS s^−1^, before they are digitally downsampled at a rate of 20 MS s^−1^.

**Figure 2:**
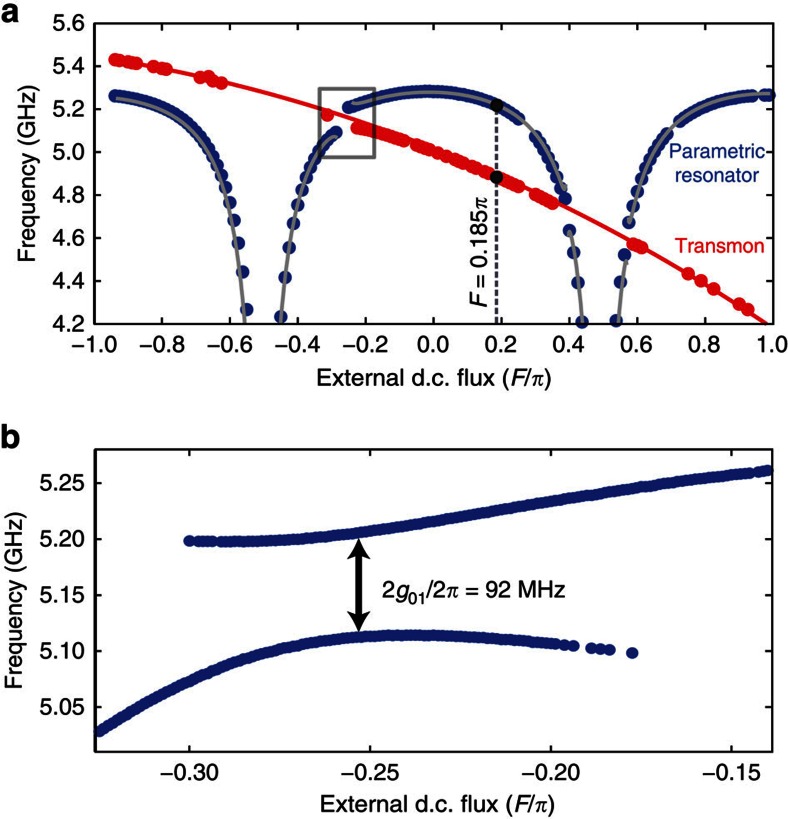
Combined resonator-qubit frequency spectra. (**a**) Qubit spectroscopy was used to map out the transmon spectrum (in red), whereas the resonator spectrum (in blue) was extracted using standard reflectometry. The solid red and grey lines are fits. The dashed grey line, at resonator flux bias *F*=0.185*π*, indicates the bias point at which we later demonstrate the read-out method. (**b**) Vacuum Rabi splitting around the flux bias point where the transmon frequency crosses that of the resonator, indicated by the grey box in **a**. The minimum frequency splitting yields a qubit–resonator coupling *g*_01_/2*π*=46 MHz.

We first characterize the transmon spectroscopically—see Fig. 2a—from which we extract the Josephson and charging energies, *E*_J_/2*π*=9.82 GHz and *E*_C_/2*π*=453 MHz, respectively. From the vacuum Rabi splitting, we extract a qubit−resonator coupling rate *g*_01_/2*π*=46 MHz—see Fig. 2b.

Next, we fit the frequency tuning curve of the resonator (with the qubit in the |0〉-state) to the relation


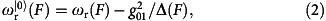


where *F*=*π*Φ_d.c._/Φ_0_ denotes the static flux bias, normalized to the magnetic flux quantum. The effective dispersive shift due to the qubit is


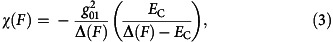


which, in turn, depends on the qubit–resonator detuning, Δ(*F*)=*ω*_a_(*F*′)−*ω*_r_(*F*′), with *F*′=*F*/8.88+0.58 representing the effective magnetic flux of the transmon. Moreover, the qubit and resonator frequency spectra are well approximated by^23^,^26^


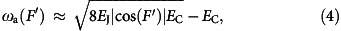



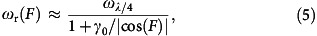


where *ω*_*λ*/4_/2*π*=5.55 GHz is the bare resonant frequency (in absence of the SQUID), and *γ*_0_=*L*_J_(*F*=0)/*L*_r_=5.3±0.1% is the inductive participation ratio between the SQUID (at zero flux) and the resonator. The solid grey and red lines in Fig. 2a are fits to equations (2) and (4), respectively.

### Single-shot qubit read-out

We now demonstrate our method for reading out the qubit with the JPO. We choose a static flux bias point *F*=0.185*π* for the resonator SQUID, corresponding to a resonant frequency 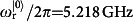 and qubit transition frequency *ω*_a_/2*π*=4.885 GHz—see dashed grey line in Fig. 2a. Consequently, the qubit–resonator detuning is Δ/2*π*=−334 MHz, and the effective dispersive shift is 2*χ*/2*π*=−7.258 MHz. We measured a Purcell-limited qubit relaxation time, *T*_1_=4.24±0.21 μs, and Ramsey free-induction decay time 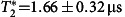
—see Methods, Supplementary Fig. 1 and Supplementary Table 1.

**Figure 3:**
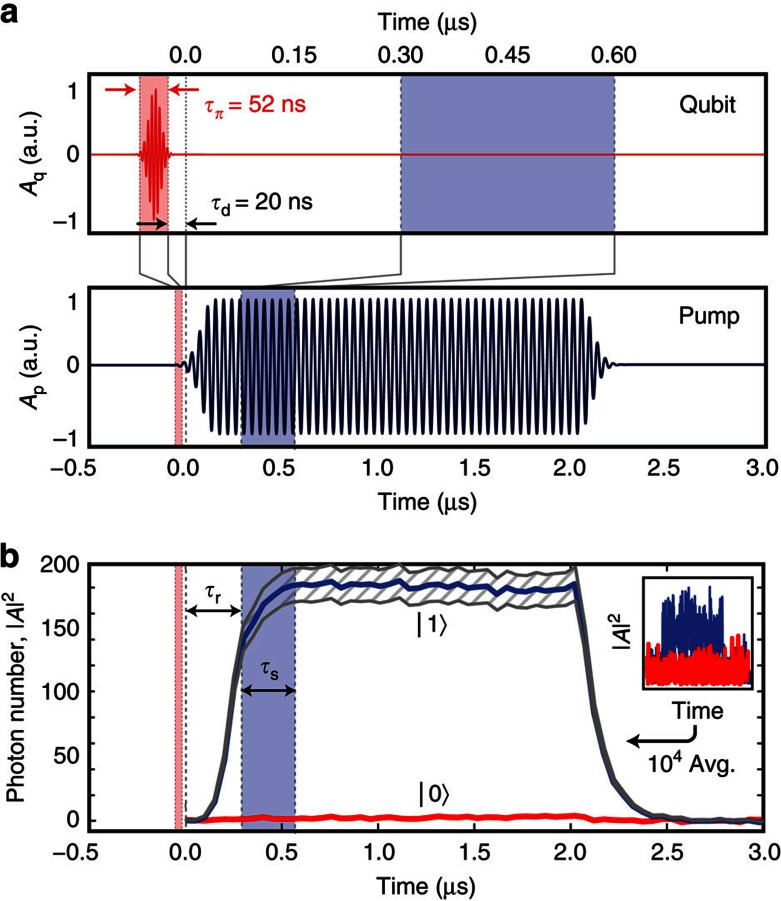
Qubit read-out by the Josephson parametric oscillator. (**a**) Pulse sequence: the qubit *π*-pulse (in red), with Gaussian edges and a plateau of duration 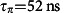, is followed by a short delay, 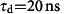, before the pump is turned on at time *t*=0. (**b**) The solid blue and red traces show the inferred photon number, |*A*|^2^, in the resonator, with and without a prior *π*-pulse on the qubit, respectively. Note that the resonator latches, once it has entered into the oscillating state, and remains there even if the qubit relaxes. The traces are the result of 10^4^ averages of the raw data; the inset shows a single instance of the raw data on the same time axis as the main plot. Before the sampling window of width 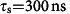, a delay 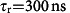 is added to avoid recording the transient oscillator response. The hatched region around the average photon number represents our uncertainty, originating from the amplifier gain calibration.

To operate the parametric oscillator as a high-fidelity qubit read-out device, we must be able to map the states of the qubit onto different states of the oscillator, which we must then clearly distinguish. We encode the qubit ground state |0〉 in the ‘quiet' state (the empty resonator) and the excited state |1〉 in the ‘populated' state of the resonator. Figure 3a shows the pulse sequence for qubit manipulation and read-out, and Fig. 3b shows the resulting output from the JPO, operated with the pump settings *δ*^|0〉^/Γ=−5.34, 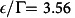.

The populated oscillator in Fig. 3b contains 185±15 photons. We obtained this estimate from a comparison between the probe-amplitude dependence of the resonant frequency and the expected photon number dependence of the Duffing shift—see Methods and Supplementary Fig. 3. This number of photons should be compared with |*A*|^2^=200±3 photons, which is the solution to equation (1) in the steady state 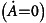.

To achieve such clear qubit-state discrimination as in Fig. 3b, we needed to make a judicious choice of flux bias point, *F*, to mitigate the effects of two nonlinear shifts of the resonant frequency^19^. The Duffing shift dominates when *F*→±*π*/2, whereas a pump-induced frequency shift dominates when *F*→0. These shifts can move the resonator away from the proper pump condition, thereby effectively restricting the output power—see Methods and Supplementary Fig. 2.

**Figure 4:**
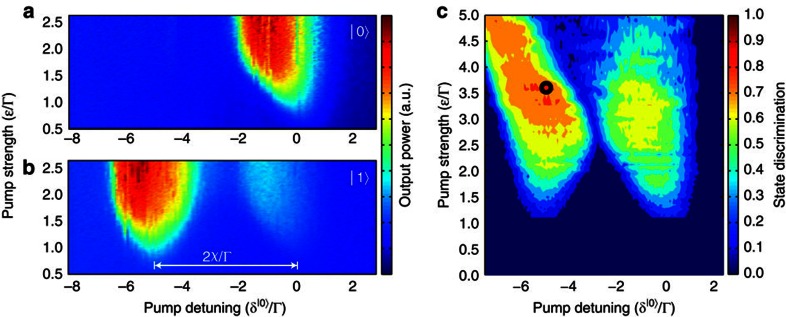
Parametric oscillations and state discrimination. Output field of the resonator when the qubit is in its (**a**) ground state |0〉 and (**b**) excited state |1〉. (**c**) Contour plot of the state discrimination within the two parametric oscillation regions. The black circle in the left region, located at *δ*^|0〉^/Γ=−5.34, 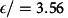, represents the bias point used throughout the rest of the analysis. The state discrimination in this point is 81.5%.

Moreover, the qubit−resonator detuning should be in the dispersive regime 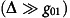, in which the qubit state controls the resonant frequency of the resonator. Yet it must yield a sufficiently large dispersive shift, *χ*>Γ (equation 3), to produce clearly distinguishable output levels, corresponding to the |0〉 and |1〉 states. For our chosen flux bias point, we identify the optimal pump settings by mapping out the parametric oscillation region as a function of pump frequency and amplitude—see Fig. 4a,b.

An interesting feature is present within the left half of Fig. 4a,b (where the populated resonator encodes |1〉). Here when the qubit is initially in the |1〉 state, the resonator latches into its oscillating state for as long as the pump is kept on, and does not transition into its quiet state when the qubit relaxes, as one might have expected. This latching is shown by the blue trace in Fig. 3b. We attribute it to the existence of a tri-stable oscillation state^17^,^21^, associated with red detuning of the above-threshold region for the |0〉 state. When the qubit relaxes, there occurs an instantaneous shift of the pseudopotential for the amplitude *A*, from bistable (with two *π*-shifted, finite-amplitude states; see Fig. 1b) to tri-stable (with one additional zero-amplitude state). The field's initial condition at the time of this shift, *A*≠0, causes the resonator to maintain its oscillating state. A separate study of this latching feature will be reported elsewhere.

**Figure 5:**
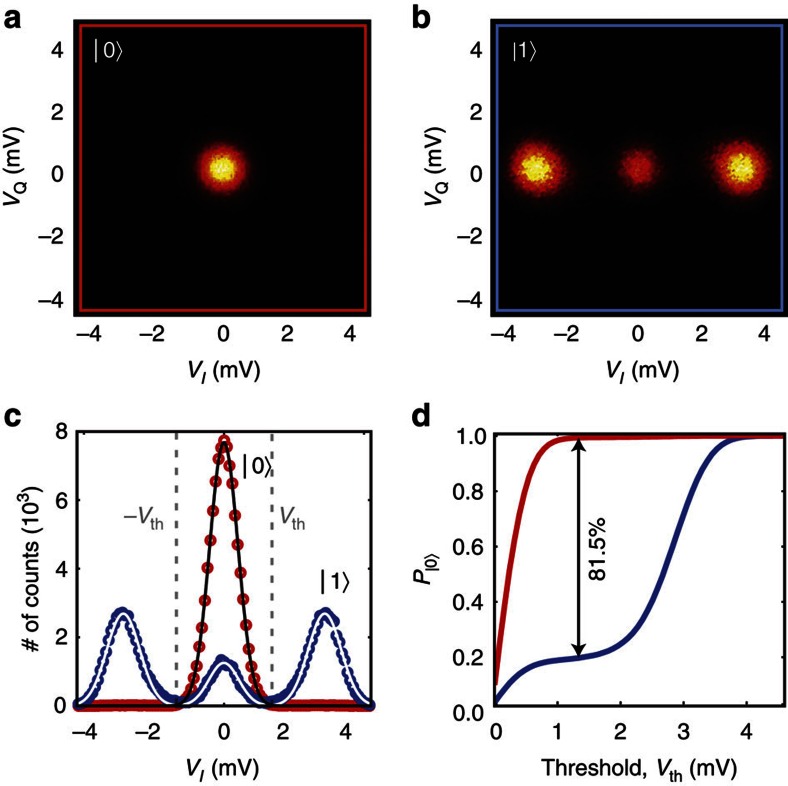
Quadrature voltage histograms of the parametric oscillator output collected after digital sampling. The pump bias point was *δ*^|0〉^/Γ=−5.34, 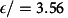. In (**a**), the qubit was in its ground state; in (**b**), a π-pulse was applied before the read-out pulse. (**c**) 1D histograms of the in-phase voltage component, *V*_I_, from the quadrature histograms in **a** and **b**. The black and white solid lines are Gaussian fits, from which we extracted a signal-to-noise ratio of 3.39. (**d**) Cumulative distribution functions, corresponding to the |0〉 and |1〉 states, obtained by sweeping a threshold voltage, *V*_th_, from the centre of the two histograms (*V*_I_=0). The maximum separation between the two S-curves yields a state discrimination of 81.5%.

We evaluate the obtainable state discrimination by collecting quadrature voltage histograms at every point within the two regions of parametric oscillations in the 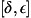-plane—see Fig. 4c. We choose the pump operation point *δ*^|0〉^/Γ=−5.34, 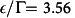, indicated by the black circle, and show the characterization in detail in Fig. 5. In this point, the state discrimination has reached a plateau around 81.5%. Each histogram in Fig. 5a,b contains in-phase (*V*_I_) and quadrature (*V*_Q_) voltage measurements from 10^5^ read-out cycles, with each measurement being the mean quadrature voltage within the sampling time 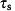 (blue window in Fig. 3). We project each of the 2D histograms onto its real axis, and thus construct 1D histograms of the *V*_I_ component—see Fig. 5c. We can then extract a SNR, 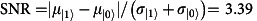, where *μ* and *σ* denote the mean value and s.d., respectively, of the Gaussians used to fit the histograms. The peak separation of the histograms gives a confidence level of 99.998% for the read-out fidelity. The peak appearing in the centre of the blue trace arises mainly from qubit relaxation before and during the read-out. We analyse this and other contributions in the next section, as well as in Supplementary Note 1 and Supplementary Fig. 4.

To extract the measurement fidelity from the histograms, we plot the cumulative distribution function of each of the two traces in Fig. 5c, by summing up the histogram counts symmetrically from the centre and outward, using a voltage threshold, *V*_th_. From these sums, we obtain the S-curves of the probability to find the qubit in its ground state as a function of the voltage threshold value—see Fig. 5d. We define the fidelity of the measurement as the maximum separation between the two S-curves.

## Discussion

To evaluate the fidelity of the read-out itself, as compared with the fidelity loss associated with qubit errors, we now present an error budget. From the histograms in Fig. 5c, we can account for 81.5% of the population, thus missing 18.5%. To understand the remaining contributions, we run a Monte Carlo simulation of the qubit population, consisting of the same number of 10^5^ read-out cycles as in the measured histograms. The simulation results are binned in the same way as the measurements, using the Gaussian fits as boundaries, and taking into account the following statistics: first, qubit relaxation and preparation errors; second, thermal population of the qubit; third, spurious switching events by *π*-radians of the oscillator phase during read-out (yielding a reduced sampled voltage); and fourth, peak separation error due to the limited SNR.

We find that the main contribution to the loss of fidelity is due to qubit relaxation before and during the read-out. From the measured relaxation time, *T*_1_=4.24±0.21 μs, we obtain a fidelity loss of 11.6±0.5%. However, this error can be reduced substantially (to <0.5%) by introducing a Purcell band-pass filter^27^,^28^,^29^ at the output of the JPO; since the qubit is detuned from the JPO, this decreases its relaxation into the 50-Ω transmission line. Such a filter would allow us to increase the resonator damping rate, Γ_0_, substantially reducing the read-out time without compromising *T*_1_. This is shown in Supplementary Note 2 and Supplementary Table 2. Note, however, that an increased resonator damping rate yields an increased width of the parametric oscillation region: consequently, the qubit–resonator coupling, *g*_01_, and detuning, Δ, need to be chosen accordingly to render a sufficiently large dispersive frequency shift.

From the simulation, we further attribute 4.5±0.3% to qubit preparation errors. Another 1.1±0.4% can be explained from thermal population of the qubit; the effective qubit temperature is *T*_q_=45±3 mK. By adding these fidelity loss contributions due to the qubit to the measured state discrimination, we can account for 81.5%+11.6±0.5%+4.5±0.3%+1.1±0.4%=98.7±1.2%.

There are also errors introduced by the parametric oscillator itself: switchings between the *π*-shifted oscillating states reduce the overall measured voltage. We performed a separate control measurement that yielded 2.4±0.5% switching probability, which translates into a maximal fidelity loss of half of that, 1.2±0.25%. The switching rate of the parametric oscillator depends on many parameters, including damping rates and bias points; this error can therefore, with careful engineering, be decreased even further. We could, however, eliminate the effect of phase-switching events using a rectifying detection scheme, for example, a diode or a field-programmable gate array, tracking the absolute value of the output field instead of its amplitude.

The last and smallest contribution to the fidelity loss is the peak separation error, which accounts for the intrinsic overlap between the histograms. However, this contribution is <0.002% for our SNR of 3.39, and can therefore be neglected. For details on the error budget analysis, see Supplementary Note 1 and Supplementary Fig. 4.

By combining the above-mentioned improvements (reduced qubit relaxation rate, optimized qubit manipulations and cooling, enhanced resonator output coupling, and rectifying data acquisition), the read-out fidelity could realistically reach ≈99.5%, limited only by the qubit relaxation.

Finally, we demonstrate that the relaxation time of our qubit is not measurably afflicted by the pump—see Methods and Supplementary Fig. 1. Our measurement scheme is, in principle, quantum nondemolition, see Supplementary Note 3; however, a proper experimental and theoretical assessment of the back-action is outside the scope of this work.

Table 1 puts our results in the context of previous work on parametric and nonlinear Josephson amplification and detection circuits.

A flux-pumped, parametric phase-locked oscillator was used as a following amplifier, also enabling sensitive qubit read-out^7^. In our work, the qubit was directly coupled with the JPO, which simplifies the experimental set-up by reducing the number of microwave components needed. Also, with a pumping amplitude below the parametric instability threshold, the flux-pumped JPA has been used to read-out one qubit^6^, as well as multiple qubits coupled with the same bus resonator^28^.

There is another way of operating our device: instead of pumping the flux at *ω*_p_≈2*ω*_r_, we can apply an alternating pump current (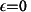, *B*(*t*≠0)), now at a frequency close to resonance, *ω*_p_≈*ω*_r_, and thereby directly modulate the phase difference, *φ*. Both methods can provide linear parametric gain on reflection of a detuned signal (*ω*_s_≠*ω*_p_/2 and *ω*_s_≠*ω*_p_, respectively). The flux-pumped JPA has a very wide frequency separation between pump tone and signal, because *ω*_s_≈*ω*_r_≈*ω*_p_/2, which is a practical advantage since it makes the resonator's entire instantaneous bandwidth available for amplification with no need to suppress or filter out the pump tone. Moreover, the *λ*/4 resonator has no mode in the vicinity of *ω*_p_ that the pump might otherwise populate.

We emphasize that there are indeed two different physical mechanisms in play, since flux and current pumping address orthogonal variables in the sense that 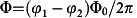 and 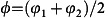, where 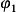 and 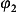 denote the gauge-invariant phase differences across the two parallel JJs. This distinction is also evident in equation (1). The parametric flux-pumping term, 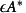, modulates the resonant frequency; it couples the resonator field amplitude and its complex conjugate, which can provide quadrature squeezing of an input signal and enables phase-sensitive parametric amplification; and for stronger modulation, there is a parametric instability threshold into the JPO regime—see Fig. 1b.

Current pumping by an input *B*(*t*), on the other hand, corresponds to an external force that directly contributes to the intra-resonator field *A* and drives its nonlinear term *α*|*A*|^2^. For zero detuning, *ω*_s_=*ω*_p_, this is the driven Duffing oscillator that has no gain (it offers no phase-sensitive amplification); for stronger driving there occurs, a dynamical bifurcation but no internal instability or parametric oscillations.

Current pumping with a moderate amplitude is used for linear amplification with the JPA^30^,^31^, which enabled, for example, the observation of quantum jumps in a qubit^5^. Current modulation is also used in the latching detection scheme of the Josephson bifurcation amplifier^9^,^10^,^14^,^32^,^33^. There, a higher-amplitude input strongly drives the Duffing nonlinearity near its bifurcation point; the two qubit states can then be mapped onto two different resonator output field amplitudes. The Josephson bifurcation amplifier was used for quantum nondemolition measurement of a qubit, and in a lumped-element resonator^11^, in which a qubit-state-sensitive autoresonance was observed in response to a frequency-chirped current drive. Yet another method is to couple the qubit with a linear resonator, which inherits a cross-Kerr nonlinearity from the qubit; current pumping of the resonator can then yield a strong output signal that depends on the qubit state^12^,^13^.

In conclusion, we have introduced a single-shot read-out technique for superconducting qubits—the JPO read-out. We demonstrated proof-of-principle operation, obtaining a bare-state discrimination of 81.5%. After correcting for known and reparable errors, this translates into an inferred read-out fidelity of 98.7±1.2%, which by implementing a rectifying detection scheme can be further increased by 1.2±0.3%. With foreseeable improvements and optimization, this device would be an attractive candidate for implementing multi-qubit read-out in the context of scalable error correction schemes. This fidelity and the read-out time are both amenable to optimization.

Our system integrates a parametric read-out mechanism into the resonator to which the qubit is coupled, substantially reducing the number of components needed to perform single-shot read-out in a circuit quantum electrodynamics architecture. Advantages offered by this read-out technique include the potential for multiplexing and scalability with no need for signal-probe inputs, additional microwave circulators, or separate parametric amplifiers. As opposed to other integrated read-out devices, our pump frequency is far outside of the resonator band and can thus easily be spectrally separated from other transition frequencies in the system.

*Note added in proof:* During the preparation of this manuscript, a new class of broad-band, Josephson parametric amplifier, the Josephson traveling wave parametric amplifier (JTWPA), was developed and published^35^.

## Methods

### Device fabrication

We fabricated our device on sapphire, using niobium for the waveguides and the transmon paddles, and shadow-evaporated aluminum for the Josephson junctions. To reduce the surface roughness before processing, the 2′′ *c* plane sapphire wafer was pre-annealed at 1,100 °C for 10 h in an atmosphere of N_2_:O_2_, 4:1, ramping the temperature by 5 °C min^−1^. The annealed wafer was then sputtered with 80 nm of Nb in a near-ultrahigh vacuum magnetron sputter. The first patterning of the sample consists of a photolithography step to define alignment marks and bond pads, deposited using electron-beam evaporation of 3 nm Ti and 80 nm Au. Next, the resonator, the transmon islands, and the pump line were defined in the Nb layer using a standard electron-beam lithography process at 100 keV, and etched using inductively coupled plasma reactive ion etching in NF_3_ gas.

The Al/AlO_*x*_/Al Josephson junctions forming the SQUIDs, used to terminate the resonator and for connecting the transmon islands, were then defined in a second electron-beam step. After exposure, the 2′′-wafer was diced into separate chips, using the exposed electron-beam resist as a protective resist. Before the first evaporation step, the surfaces of the Nb films where cleaned using *in situ* Ar-ion milling inside of the Plassys evaporator. However, due to the substantially different regimes of critical currents, *I*_0_, required for the Josephson junction of the transmons and the parametric resonator, two sequential evaporations and oxidations were performed within the same vacuum cycle by rotating a planetary aperture mounted inside the evaporator load-lock, effectively shielding one half of the sample at the time. Finally, a post-deposition ashing step was performed to clean the surfaces from organic residues.

### Finding the parametric oscillation threshold

It is hard to experimentally find the parametric oscillation threshold with good precision, when only considering the parametric oscillation region, Fig. 4a, whose observed shape gets smeared by the amplified vacuum noise. In this section, we present an alternative method using a weak probe signal: we probe the parametrically amplified response as we sweep the pump amplitude across the instability threshold.

We apply a probe signal on resonance, 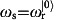, while applying a detuned pump signal, such that (*ω*_p_−2*ω*_s_)/2*π*=100 kHz. The signal then undergoes degenerate, phase-preserving parametric amplification (red trace in Supplementary Fig. 5), while the parametric oscillations are cancelled out since we measure the average amplitude of the field. The parametric amplification has maximum gain just at the threshold. We plot the magnitude of the reflected signal as a function of the pump power (at the generator), yielding an oscillation threshold *P*_th_=−10.8 dBm, as indicated by the dashed red line. As a comparison, we measure the output power of parametric oscillation, for *ω*_p_−2*ω*_r_=0 and *B*(*t*)=0—see the blue trace.

### Limits of the parametric oscillation amplitude

As briefly discussed in the main text, there are two nonlinear effects that move the resonator away from its pump condition, by means of their associated frequency shifts^19^,


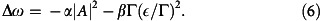


The Duffing shift dominates near flux bias *F*=±*π*/2; the Duffing parameter is approximated as


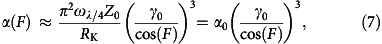


where *Z*_0_=50 Ω is the resonator's characteristic impedance and *R*_K_=*h*/*e*^2^ is the quantum resistance.

The pump-induced frequency shift dominates near *F*=0; it is approximated as


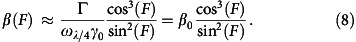


The resonator's frequency tuning versus *F*, equation (5) in the main text, is shown in Supplementary Fig. 3a, for the parameters of our device, and equations (7) and (8) are plotted in Supplementary Fig. 3b. This figure illustrates that it is essential to bias the system far enough away from the limiting points, *F*=0 or *π*/2, such that neither frequency shift pulls the resonator too far from its pump condition, thereby severely limiting the attainable output power.

The steady-state solution of equation (1) in the main text yields an analytic expression for the expected number of photons within the region of parametric oscillations,


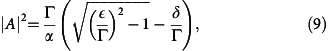


which, for our analysed bias point, amounts to 200±3 photons in the resonator. From this number, we obtain a Duffing shift −*α*|*A*|^2^/2*π*≈−5.4±0.3 MHz (for *α*/2*π*=27±1.5 kHz per photon) and a pump-induced frequency shift 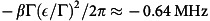 (for *β*=(7.5±0.1) × 10^−3^).

The parameter *β* has the effect of skewing the parametric oscillation region, yielding an expression for the thresholds plotted in Fig. 1b,


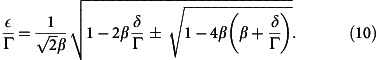


### Calibration of attenuation and gain via the Duffing nonlinearity

In this section, we present how we calibrated the gain of the amplifier chain, using the photon-number-dependent frequency shift of the Duffing oscillator, −*α*|*A*|^2^, which we recall from the previous section. The frequency of the resonator as a function of input probe power takes the following form,


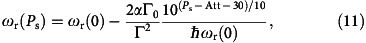


where *ω*_r_(0) denotes the resonant frequency with zero photons in the resonator, Γ_0_ and Γ are the external and total loss rates, respectively, and *α* is the Duffing frequency shift per photon—recall equation (7). Using equation (11), we can fit the extracted resonant frequencies as a function of input probe power at different flux bias points, *F*, with the attenuation, Att, as the only fitting parameter (since *α* can be extracted separately by fitting *ω*_*λ*/4_ and *γ*_0_—recall equation (5)). This is shown in Supplementary Fig. 2, where the data for five different flux bias points are fitted to attenuations presented in Supplementary Table 3. From these values, we obtain an average attenuation, 〈Att〉=127.5 dB, which can be compared with the installed 120 dB, indicating that we have a cable loss of 7.5 dB at the measurement frequency.

Moreover, from the same measurement, we can also obtain an estimate for the gain of the amplifier chain by assuming that all the signal gets reflected when it is far off resonance with the resonator, that is, reflection coefficient |*S*_11_|^2^=1. Then, the gain is obtained from the relation


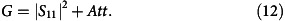


For the five gain estimates presented in Supplementary Table 3, we obtain a gain of *G*=81.0±0.37 dB, at our given bias point. The error bars for this gain estimation has two origins: ±0.17 dB from the residual of the linear fit to the gain values presented in Supplementary Table 3, and another ±0.2 dB from the gain drift over time, which can be compared with our 91 dB of installed amplification.

### Calibration of the resonator photon number

From the obtained calibration of the gain of our amplifier chain, *G*, we can now calculate the conversion factor between our measured power on the digitizer and the number of photons in the resonator, using the following relation,


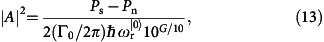


where *P*_s_ and *P*_n_ denote our signal and noise power levels, respectively. We demonstrate this for Fig. 3b, where the resonator is probed at a frequency 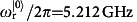. The external damping rate is Γ_0_/2*π*=1.02 MHz, and we calculate the background power level from the end of the trace (when the pump is off). From the obtained SNR, the number of added noise photons can be estimated accordingly, |*A*|^2^/SNR^2^=16.1±1.3.

### Quantum coherence and read-out nondestructiveness

To study how the parametric pump strength affects the qubit's relaxation time, we here present coherence measurements for the transmon. First, we calibrate a qubit pulse duration corresponding to a *π*-pulse, using a Rabi measurement, where the pulse duration time is swept, for a fixed pulse amplitude. From the fit in Supplementary Fig. 1a, a pulse length of 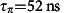 was obtained, and the Rabi decay time was *T*_rabi_=2.53±0.15 μs. The histograms corresponding to the first 0.5 μs are plotted in Supplementary Fig. 1b, using the same projective technique as for the histograms in Fig. 5c in the main text. Finally, we perform a set of *T*_1_ measurements for different pump amplitudes 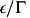, and compare these with traditional reflection read-out, where we apply a weak resonant probe signal, but no pump 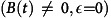. The fits to the relaxation times suggest that our read-out is not any more destructive to the quantum state of the transmon than the traditional read-out technique is. We note, however, that our extracted relaxation time is limited by the Purcell effect, yielding *T*_1_≈[2Γ_0_(*g*_01_/Δ)^2^]^−1^=4.11 μs. Also see Supplementary Note 3.

## Additional information

**How to cite this article:** Krantz, P. *et al*. Single-shot read-out of a superconducting qubit using a Josephson parametric oscillator. *Nat. Commun.* 7:11417 doi: 10.1038/ncomms11417 (2016).

## Supplementary Material

Supplementary Information Supplementary Figures 1-5, Supplementary Tables 1-4, Supplementary Notes 1-3 and Supplementary References

## Figures and Tables

**Table 1:** Overview of different modes of operation for the various Josephson amplification and detection schemes.

**Device**	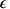	***B***_**s**_	***B***_**p**_	**# Modes**	**Reference**
JPO^(^*^)^	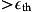	0	0	1	This work
JPA	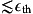	≠0	0	1	6
JPA	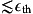	≠0	0	Multimode	34
PPLO	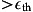	≠0	≠0	1	7
JPA	0	≠0	≠0	1	5
JBA^(^*^)^	0	0	≠0	1	10
JPC	0	≠0	≠0	2	8

JBA, Josephson bifurcation amplifier; JPO, Josephson parametric oscillator; PPLO, parametric phase-locked oscillator

The variables refer to equation (1), where 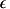 denotes the flux-pumping amplitude (at *ω*_p_≈2*ω*_r_), and *B*_s_ and *B*_p_ denote a.c. signal and pump amplitudes, respectively (at *ω*_p_≈*ω*_r_). The two read-out methods marked with an asterisk (*) have the qubit directly integrated with the detector, whereas the other devices are used as following amplifiers.
