# Supplementary material for: Single-shot read-out of a superconducting qubit using a Josephson parametric oscillator
Source: Nat Commun. 2016 May 9;7:11417. doi: 10.1038/ncomms11417 (PMC4865746; doi:10.1038/ncomms11417)
Supplement: Supplementary Information — Supplementary Figures 1-5, Supplementary Tables 1-4, Supplementary Notes 1-3 and Supplementary References [file ncomms11417-s1.pdf]

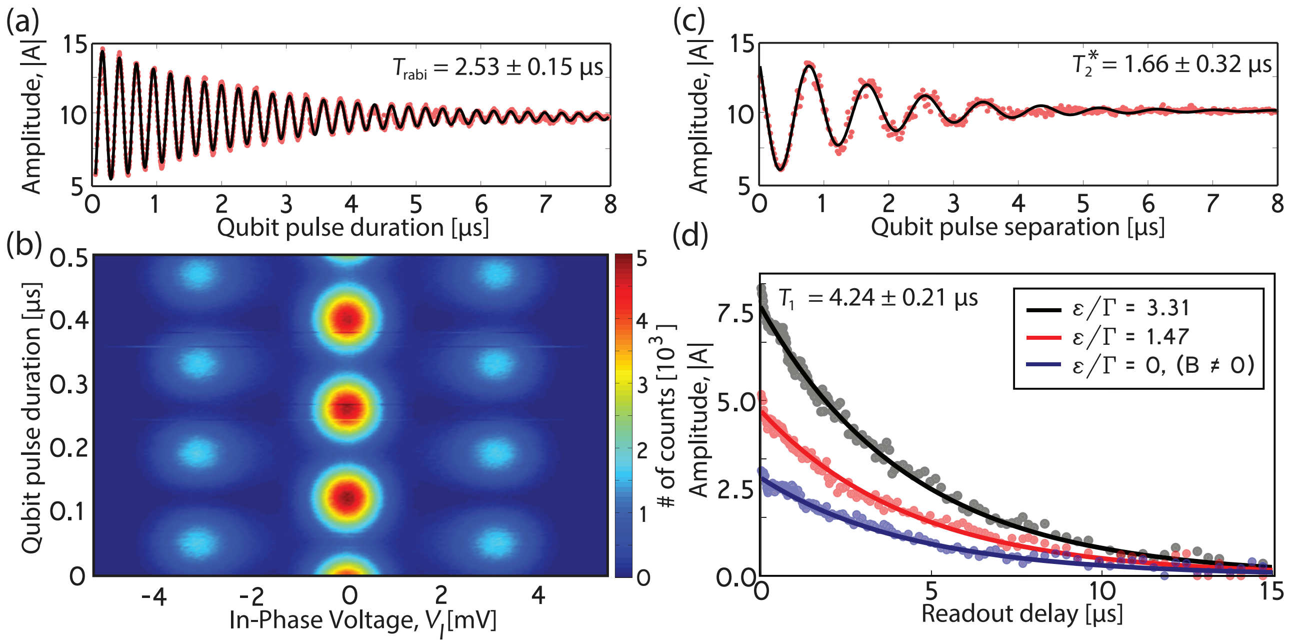

Supplementary Figure 1. Qubit coherence measurements. **(a)** Rabi oscillations expressed in field amplitude, $|A|$, (number of photons)$^{1/2}$. **(b)** Histogram representation of the first $0.5\,\mu$s of the Rabi oscillations measured in (a). **(c)** Ramsey free-induction decay (separation time between two $\pi/2$-pulses), T_2^*. **(d)** T_1 relaxation times for two different resonator pump amplitudes during readout, $\epsilon/\Gamma = 3.31$ (black) and 1.47 (red), as well as a reference measurement using a weak resonant probe signal through the coupling capacitor, $B(t) \neq 0$ (blue). The solid lines are exponential fits, yielding relaxation times presented in Supplementary Table 1.

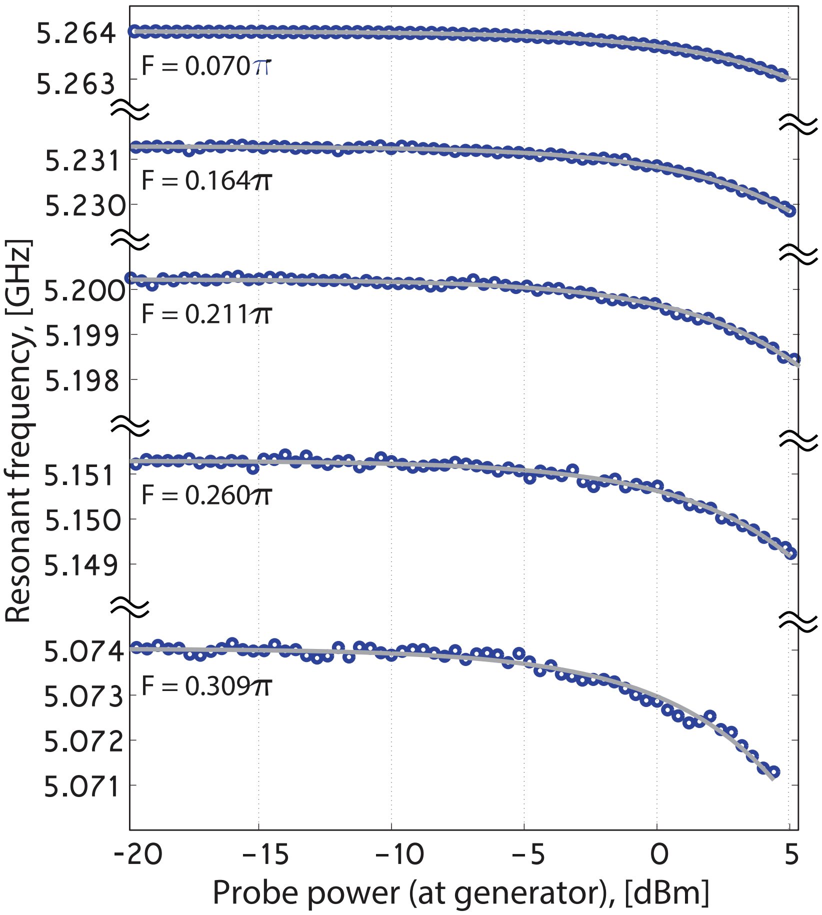

Supplementary Figure 2. Extraction of the non-linear Duffing frequency shift as a function of probe power. The grey solid lines are fits to Eq. (11), indicating an increased shift as the resonant frequency in Supplementary Fig. 3(a) moves further down on the tuning curve. For every point, we fit the attenuation of the input line, Att — see Supplementary Table 1.

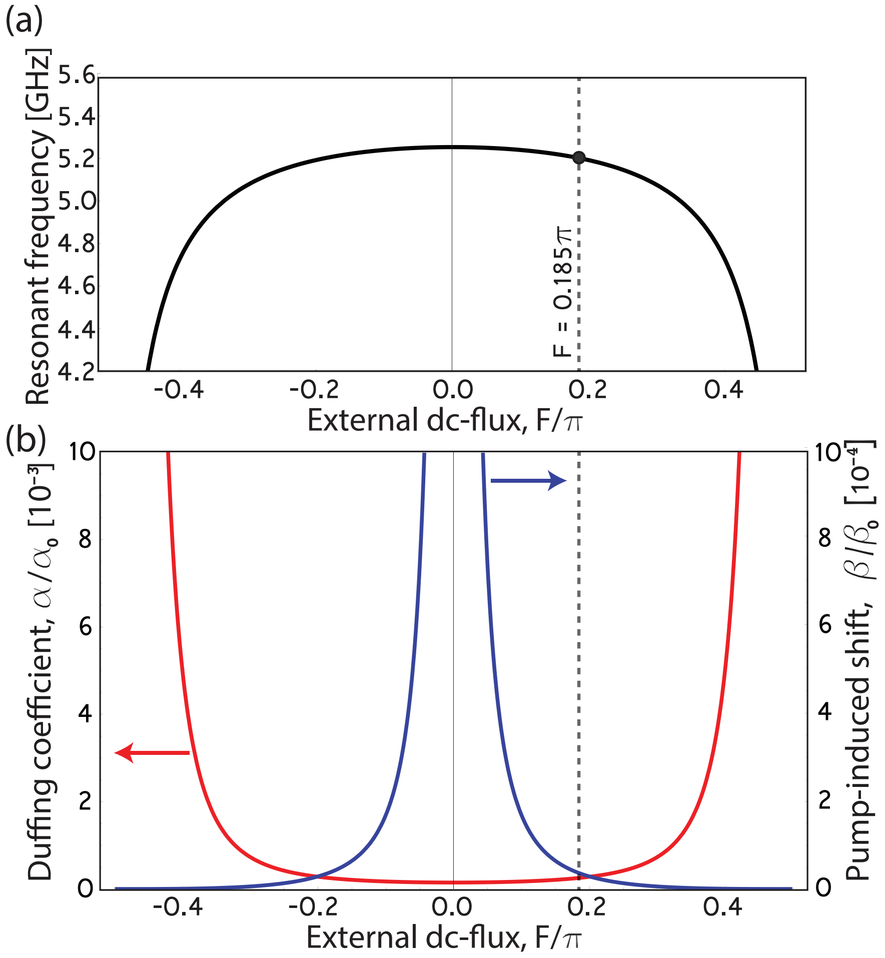

Supplementary Figure 3. Flux-dependence of JPO nonlinearities. **(a)** Resonator frequency dependence on magnetic flux for the bare resonant frequency, $\omega_{\lambda/4}/2\pi = 5.55\,\mathrm{GHz}$, and inductive participation ratio, $\gamma_0 = 5.3 \pm 0.1\,\%$. The dashed line indicates flux bias point, $F = 0.185\,\pi$, used throughout this analysis. **(b)** Duffing and pump-induced nonlinearities as functions of the magnetic flux bias, F.

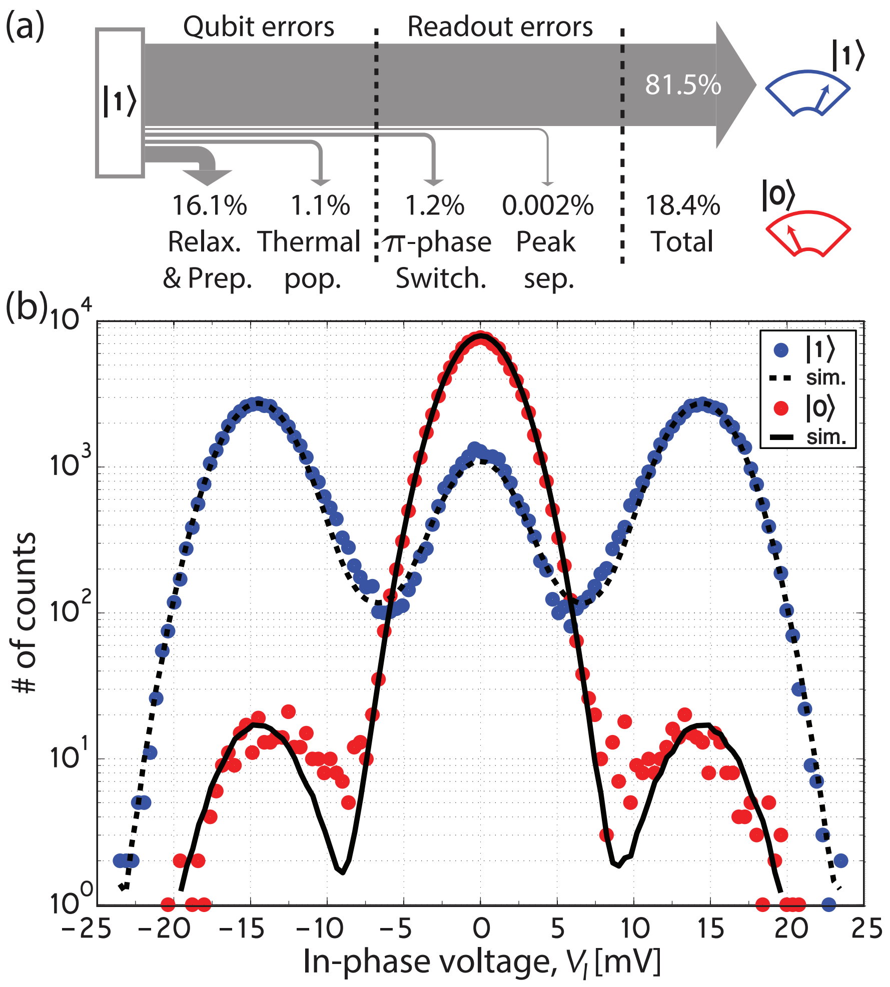

Supplementary Figure 4. Schematics, and outcome of qubit population Monte Carlo simulation. **(a)** Schematics of the different contributions going into the simulation. The errors are divided into qubit errors and readout errors. **(b)** The solid red and blue dots are the histogram bins from Fig. 5(c), here plotted on a logaritmic scale. The solid and dashed black lines represent the outcome of the simulation of the different error contributions.

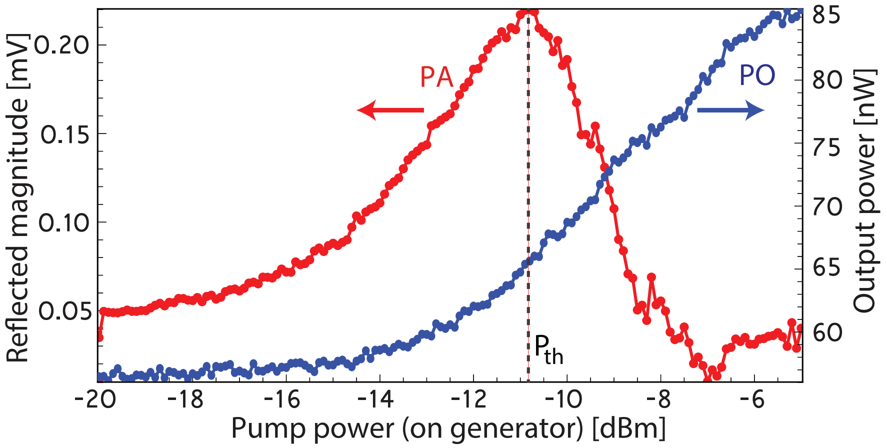

Supplementary Figure 5. Extraction of parametric oscillation threshold. Reflected magnitude of non-degenerate parametric amplification (PA, red trace and left y-axis) and power of parametric oscillations (PO, blue trace and right y-axis) as a function of pump power across the instability threshold (dashed line). From the parametric amplification trace, an oscillation threshold, $P_{\mathrm{th}} = -10.8\,\mathrm{dB}$ was obtained.

Readout type	Pump amplitude ϵ/Γ	Relaxation time T_1 [μs]
JPO (black)	3.31	4.24±0.21
JPO (red)	1.47	4.18±0.19
Refl. (blue)	0	4.32±0.22

Supplementary Table 1. Relaxation time, T_1, for two different pump amplitudes as compared with a reflection reference measurement.

	Purcell filter Q_f	Qubit-resonator detuning $\Delta/2\pi$ [MHz]	External quality factor Q_{ext}	Total damping rate $\Gamma/2\pi$	Ring-up time τ_r [ns]	Sampling time τ_s [ns]	Relaxation time T_1 [μs]	Predicted fidelity loss F_L [%]
	-	-334	2554	1.02	206	300	4.11	8.51[a]
**(a)**	-	-334	1000	2.61	101	46	1.61	8.00
	-	-334	500	5.21	51	12	0.80	7.96
	200	-334	2554	1.02	206	300	99.0	0.37
**(b)**	200	-334	1000	2.61	101	46	38.8	0.34
	200	-334	500	5.21	51	12	19.4	0.34
	200	-500	2554	1.02	206	300	323	0.11
**(c)**	200	-500	1000	2.61	101	46	126	0.11
	200	-500	500	5.21	51	12	63	0.07
	200	-500	2554	1.02	206	300	50	0.73
**(d)**	200	-500	1000	2.61	101	46	50	0.27
	200	-500	500	5.21	51	12	50	**0.13**

[a] Note that the π-pulse duration, delay time, and ring-up time, are all shorter here compared with the experiment, yielding reduced fidelity loss. For our experiemental values, $\tau_\pi = 52\,\text{ns}$, $\tau_d = 20\,\text{ns}$, $\tau_r = 300\,\text{ns}$, and $\tau_s = 300\,\text{ns}$, we obtained $F_L = 11.6\,\%$.

Supplementary Table 2. Theoretical comparison of readout fidelity losses with and without a Purcell bandpass filter, for three different external damping rates of the JPO. **(a)** Without Purcell filter. **(b)** With Purcell filter and maintaining the qubit-resonator detuning $\Delta/2\pi = -334\,\text{MHz}$ from the experiment. **(c)** Increased detuning to $\Delta/2\pi = -500\,\text{MHz}$. **(d)** Assuming a realistically attainable qubit coherence time, $T_1 = 50\,\mu\text{s}$ (limited by other channels than Purcell decay).

dc-flux bias	F/π	0.070	0.164	0.211	0.260	0.309	
Attenuation	Att	-126.9	-127.1	-127.5	-127.8	-127.9	dB
Gain	G	80.3	80.6	81.4	81.4	82.0	dB

Supplementary Table 3. Extracted attenuation for the five different dc-flux bias points used for fitting the Duffing nonlinearity from Eq. (11) — see Supplementary Figure 2. The corresponding gain values were obtained from Eq. (12).

Sec.	Error source	Comment	F_{m} [%]
(i)	Qubit relaxation	T_1 decay	11.6±0.5
	Qubit preparation	Combined init. errors	4.5±0.3
(ii)	Thermal population	$T_q = 45{\pm}3\,\mathrm{mK}$	1.1±0.4
(iii)	Switching events	Property of oscillator	1.2±0.3
(iv)	Peak separation	Histogram overlap	<0.002
	**Total fidelity loss**		$\mathbf{18.4 \pm 1.5}$
	Measured fidelity	Derived from S-curves	81.5
	**Explained fidelity**		$\mathbf{99.9 \pm 1.5}$

Supplementary Table 4. Summary of the error budget analysis, starting out from the state discrimination of 81.5%.

SUPPLEMENTARY NOTE 1: FIDELITY ERROR BUDGET

In this note, we present an analysis of how we can distinguish between the different sources of the reduction in measurement fidelity. We calculate the fidelity from the maximum separation of the S-curves of the histogram data, representing the qubit in the ground and excited states — see Fig. 5(d). To quantify the missing fidelity, we here present an error budget where we discuss five different sources of fidelity loss. In our system, there are losses that are due to the qubit and those that are due to the parametric oscillator. The different loss contributions, discussed in this section, are presented in Supplementary Table 2.

(i) Qubit relaxation and preparation errors

The largest contribution to the reduction of fidelity is associated with the qubit relaxation time, $T_1 = 4.24 \pm 0.21\,\mu$s. The time scales for the readout are $\tau_\pi = 52\,$ns (π pulse), $\tau_d = 20\,$ns (delay before readout), $\tau_r = 300\,$ns (oscillator rise time), and $\tau_s = 300\,$ns (sampling time of the parametric oscillator). This means that the pre-sampling time (including the π-pulse) equals $\tau_{\text{pre}} = \tau_\pi + \tau_d + \tau_r = 372\,$ns, which, together with the relaxation during the readout yields a fidelity loss of $1 - \exp\left(-(\tau_{\text{pre}} + \tau_s/2)/T_1\right) = 11.6 \pm 0.5\,\%$. Here, we count only half of the sampling time, τ_s, since a qubit decay event occuring during the second half of the sampling time still ends up on the right side of the voltage threshold, V_{th}.

Next, to rule out dark or missed counts due to spurious oscillation events as well as non-triggered oscillations, we performed two separate measurements on the JPO (without involving the qubit) in which statistics of raw data indicated the absence of both these events. The Monte Carlo simulation outcome yields an upper limit for our qubit preparation error of 4.5±0.3 %, thus summing up to 11.6±0.5 % + 4.5±0.3 % = $16.1 \pm 0.8\,\%$.

(ii) Thermal population of the qubit

Already from Fig. 5(c) in the main text, we see that the thermal population of the qubit is small. However, from the ground-state histograms in the logaritmic plot, we can make an estimate of the qubit temperature using the Boltzmann distribution function, $1/\exp[\hbar\omega_a/(k_B T_q)]$, with a temperature $T_q = 45 \pm 3\,$mK, and assuming that the qubit can only populate its two lowest energy levels. By summing up the counts outside of a voltage threshold of $V_{\text{th}} = \pm 1.8\,$mV, we arrive at a $0.55 \pm 0.2\,\%$ probability of finding the qubit in its excited state. However, since this will act on both the ground and excited states, it renders a doubled fidelity loss contribution of 1.1±0.4 %.

(iii) Switching events during the readout cycle

Each readout count is obtained, in the digitizer, by averaging the downconverted voltage during the sampling window of $\tau_s = 300\,$ns; see Fig. 3. There is a certain probability that a π-phase switching event of the parametric oscillator occurs during this time. Such switching events cause smearing of the histograms towards the center, which is most pronounced for the blue histogram in Fig. 4 (since it consists mainly of oscillating counts). To investigate this source of error, we analyzed in more detail the raw data for 10^3 readout cycles. After disregarding non-oscillating traces (which represent events of qubit relaxation prior to the readout), we found that 2.4±0.5 % of the oscillating traces contained switching events. The error bar on the extracted switching rate come from assuming a binominal distribution of the $n = 1000$ measurements, and a switching probability $p = 2.4\,\%$, yielding a standard deviation, $\sigma/n = \sqrt{np(1-p)}/n \approx 0.5\,\%$. This translates to a fidelity loss of 1.2±0.25%, since half of the switching events give rise to the correct measurement outcome regardless of the switching event due to the fact that these still end up on the correct side of the threshold. This error can, however, be eliminated by implementing a rectifying detection scheme, using for instance an FPGA — making π and $-\pi$ indistinguishable.

(iv) Peak separation

To ensure that the peak separation between the histograms is sufficient to fully distinguish between the readout events, we did a full region map of the fidelity as a function of pump frequency and power, see Fig. 4(b). In addition, this map serves as a guide for optimizing the readout contrast. The indicated spot shows the bias point $\delta^{|0\rangle}/\Gamma = -5.34, \epsilon/\Gamma = 3.56$, used throughout this error-budget analysis.

This bias point is positioned on a plateau of fairly constant fidelities. As we move down in pump strength, the fidelity is reduced. This can be understood from the reduction of the signal-to-noise ratio, which results in an overlap between the Gaussian histograms. The same is true for the opposite encoding, found within the right region, where the populated resonator encodes qubit state $|0\rangle$. There, the SNR is insufficient for full state discrimination. At the bias point used, the pump is sufficiently strong for a negligible probability of non-oscillations, given that the qubit has been properly initialized. We can extract this overlap by fitting two Gaussians to both oscillating states, and one Gaussian to the center peak. From these fits, we extract a peak separation error of $< 0.002\%$.

SUPPLEMENTARY NOTE 2: REDUCTION OF FIDELITY LOSS AND READOUT TIME USING A PURCELL FILTER

From the error budget presented in Supplementary Note 1, we conclude that the largest contribution to the loss of readout fidelity is associated with qubit relaxation prior to and during readout. Fortunately, this error can be substantially suppressed by implementing a bandpass (Purcell) filter at the JPO output [1, 3, 4]. Here we present an estimate of the expected performance.

A Purcell filter would improve our readout fidelity in two ways: (i) it would increase the Purcell limitation on the qubit relaxation time, and (ii), it would allow for an increase of the resonator external damping rate. The latter both reduces the resonator ring-up time, τ_r, and increases the signal-to-noise ratio, allowing us to reduce the sampling time, τ_s. In this Note, we elaborate on the details of these readout improvements.

(i) Increase of the Purcell limitation

Let us first look at three different values of the external damping rate, $\Gamma_0/2\pi$, and how an added bandpass filter, centered around ω_r, with a quality factor $Q_f = 200$, changes the qubit relaxation time. The Purcell-limited relaxation time is given by

$$T_1 = \frac{1}{2\Gamma_0}\left(\frac{\Delta}{g_{01}}\right)^2, \tag{1}$$

which for our resonator and bias point results in $T_1 = 4.11\,\mu$s – see the first row in Supplementary Table 2. By adding a filter of bandwidth ω_r/Q_f, the effective damping of the qubit into the 50-Ω load decreases without compromising the readout time of the resonator, modifying Eq. (1) into

$$T_1^f = \frac{1}{2\Gamma_0}\left(\frac{\Delta}{g_{01}}\right)^2\left(\frac{\omega_r}{\Delta+\omega_r}\right)\left(\frac{2\Delta}{\omega_r/Q_f}\right). \tag{2}$$

In Supplementary Table 2, we make a theoretical comparison of the fidelity loss due to T_1 relaxation, for external quality factors $Q_{\text{ext}} = 2554$, 1000, and 500. The calculations use a shorter measurement time (see section (ii) here below), but otherwise the same parameters as in our experiment: resonator frequency, $\omega_r/2\pi = 5.212\,$GHz, qubit transition frequency, $\omega_a/2\pi = 4.885\,$GHz, qubit-resonator coupling, $g_{01}/2\pi = 46\,$MHz, and qubit-resonator detuning, $\Delta/2\pi = -334\,$MHz. We do the calculations with (a) and without (b) an added Purcell filter, and then for a greater qubit–resonator detuning (c), and finally (d) for a realistically achievable transmon relaxation time T1 = 50 us (limited by other channels of decoherence).

The conclusion is that the Purcell filter substantially suppresses the fidelity loss due to relaxation, well below 1 %, see the last row in the table.

(ii) Decrease of the external quality factor

The enhanced resonator damping rate would, for a linear resonator, yield a reduced ring-up time $\tau_r = 2Q_{\text{ext}}/\omega_r$. The nonlinearity of the JPO leads to saturation of the intra-resonator field, at a time scale related to Q_{ext}. By numerically solving the time-dependent Eq. (1) in the main text, we achieve agreement with the measurements, allowing us to estimate τ_r for various values of Q_{ext}. We note that τ_s can realistically be decreased by 4 times – see Supplementary Table 2.

Moreover, the reduced Q-value enables a reduction in the sampling time while maintaining a sufficient SNR. Recalling the steady-state solution for the equation of motion (Eq. (9) from the Methods section), we see that for a constant normalized pump strength, ϵ/Γ, and zero pump-detuning, $\delta/\Gamma = 0$, the intra-resonator field amplitude, A, scales with the square root of the damping rate,

$$|A| = \sqrt{\frac{\Gamma}{\alpha}\sqrt{\left(\frac{\epsilon}{\Gamma}\right)^2 - 1}}. \tag{3}$$

The output signal amplitude (in absence of an input probe field) can be expressed in terms of A,

$$|C| = \sqrt{2\Gamma_0}\,|A|\,. \tag{4}$$

For an overcoupled resonator, $\Gamma \approx \Gamma_0$ and $Q \approx Q_{\rm ext}$, Eq. (4) can be written in terms of $Q_{\rm ext}$,

$$|C| \approx \Gamma_0\sqrt{\frac{2}{\alpha}\sqrt{\left(\frac{\epsilon}{\Gamma}\right)^2 - 1}} = \frac{\omega_r}{Q_{\rm ext}}\sqrt{\frac{1}{2\alpha}\sqrt{\left(\frac{\epsilon}{\Gamma}\right)^2 - 1}}. \tag{5}$$

The detected signal increases inversely proportionally to the external Q-value. To keep the same signal-to-noise ratio, while decreasing $Q_{\rm ext}$, we can thus afford to reduce the measurement time substantially. Reducing the external Q-value by a factor 5 to 500 yields a sampling time $\tau_s = 300/25 = 12\,$ns. Using a π-pulse of duration $\tau_\pi = 10\,$ns, the pre-sampling time becomes $\tau_{\rm pre} = \tau_\pi + \tau_d + \tau_r = 10 + 0 + 51\,$ns, and the total measurement time is reduced to $\tau < 100\,$ns, which is comparable with state-of-the-art readout schemes based on following parametric amplifiers. Consequently, the loss of fidelity due to qubit relaxation becomes,

$$F_L = 1 - \exp\left[-(\tau_{pre} + \tau_s/2)/T_1\right] \approx 0.13\,\%, \tag{6}$$

see the last row of Supplementary Table 2.

The readout time could be reduced even further by optimising the nonlinearity, α, and the pump amplitude, ϵ, and by using an even lower-noise amplifier [5]. We can increase the dispersive shift, χ, to $>\ 50\,$MHz by using the qubit straddling regime [6], where the qubit-resonator detuning is such that $0 < \Delta < E_c$.

SUPPLEMENTARY NOTE 3: QUANTUM NON-DEMOLITION

The joint qubit-resonator system can be described, in the laboratory frame, by the Jaynes-Cummings Hamiltonian in the dispersive approximation,

$$\hat{H}_{\mathrm{disp}}/\hbar = \omega_r \hat{a}^\dagger \hat{a} + \frac{\alpha}{2}\left(\hat{a}^\dagger \hat{a}\right)^2 - \frac{\omega_a + \chi}{2}\hat{\sigma}_z - \chi \hat{\sigma}_z \hat{a}^\dagger \hat{a}, \tag{7}$$

where $\hat{a}$ and $\hat{a}^\dagger$ denote the bosonic single-mode annihilation and creation operators, respectively, and $\hat{\sigma}_z$ is a Pauli matrix. Our measurement protocol is, in principle, quantum nondemolition (QND), since the qubit-field interaction term commutes with the free qubit Hamiltonian [7]. This is no different from the usual measurement principle in circuit-QED with a qubit coupled to a linear resonator [8].

There are two main differences between our JPO readout and conventional dispersive readout: First, our system has an additional term describing the photon-number dependent, nonlinear Duffing shift, $\frac{\alpha}{2}(\hat{a}^\dagger \hat{a})^2$; however, this term is small due to the weak nonlinearity, $\alpha \ll \Gamma_0$, and the dispersive approximation still holds. Second, our resonator photon number, $|A|^2 = 185 \pm 15$, is higher than what is typical for conventional dispersive readout, although it is similar to the number reported by other groups [1, 9]. A theoretical and experimental investigation of the QND-ness of our measurement method is outside the scope of this paper.

[1] E. Jeffrey *et al.*, Fast accurate state measurement with superconducting qubits, Phys. Rev. Lett. **112**, 257402 (2014).
[2] P. Krantz *et al.*, Investigation of nonlinear effects in Josephson parametric oscillators used in circuit quantum electrodynamics, New J. Phys. **15**, 105002 (2013).
[3] M. D. Reed *et al.*, High-fidelity readout in circuit quantum electrodynamics using the Jaynes-Cummings nonlinearity, Phys. Rev. Lett. **105**, 173601 (2010).
[4] E. A. Sete, J. M. Martinis, and A. N. Korotkov, Quantum theory of a bandpass Purcell filter for qubit readout, Phys. Rev. A **92**, 012325 (2015).
[5] J. Schleeh *et al.*, Ultralow-Power Cryogenic InP HEMT With Minimum Noise Temperature of 1 K at 6 GHz, IEEE Elec. Dev. Lett. **33**, 664-666 (2012).
[6] J. Koch *et al.*, Charge-insensitive qubit design derived from the Cooper pair box, Phys. Rev. A **76**, 042319 (2007).
[7] V. B. Braginsky, Quantum nondemolition measurements: The route from toys to tools, Rev. Mod. Phys. **68**, 1 (1996).
[8] A. Blais, R.-S. Huang, A. Wallraff, S. M. Girvin, and R. Schoelkopf, Cavity quantum electrodynamics for superconducting electrical circuits: An architecture for quantum computation, Phys. Rev. A **69**, 062320 (2004).
[9] F. Mallet *et al.*, Single-shot qubit readout in circuit quantum electrodynamics, Nat. Phys. **5**, 791-795 (2009).
